# Biomarker of Neuroinflammation in Parkinson’s Disease

**DOI:** 10.3390/ijms23084148

**Published:** 2022-04-08

**Authors:** Tsai-Wei Liu, Chiung-Mei Chen, Kuo-Hsuan Chang

**Affiliations:** 1 Linkou Medical Center, Department of Neurology, Chang Gung Memorial Hospital, Tauoyan 333, Taiwan; pupucarliu@cgmh.org.tw (T.-W.L.); cmchen@cgmh.org.tw (C.-M.C.); 2 School of Medicine, Chang Gung University, Taoyuan 333, Taiwan

**Keywords:** Parkinson’s disease, inflammation, microglia, biomarker, interleukin (IL)-1β, IL-2, IL-6, IL-10, Tumor necrosis factor (TNF)-α, normal T cell expressed and presumably secreted (RANTES), high-sensitivity c-reactive protein (hsCRP)

## Abstract

Parkinson’s disease (PD) is caused by abnormal accumulation of α-synuclein in dopaminergic neurons of the substantia nigra, which subsequently causes motor symptoms. Neuroinflammation plays a vital role in the pathogenesis of neurodegeneration in PD. This neuroinflammatory neurodegeneration involves the activation of microglia, upregulation of proinflammatory factors, and gut microbiota. In this review, we summarized the recent findings on detection of PD by using inflammatory biomarkers, such as interleukin (IL)-1β, IL-2, IL-6, IL-10, tumor necrosis factor (TNF)-α; regulated upon activation, normal T cell expressed and presumably secreted (RANTES) and high-sensitivity c-reactive protein (hsCRP); and radiotracers such as [11C]PK11195 and [18F]-FEPPA, as well as by monitoring disease progression and the treatment response. Many PD-causing mutations in *SNCA*, *LRRK2*, *PRKN*, *PINK1*, and *DJ-1* are also associated with neuroinflammation. Several anti-inflammatory medications, including nonsteroidal anti-inflammatory drugs (NSAID), inhibitors of TNF-α and NLR family pyrin domain containing 3 (NLRP3), agonists of nuclear factor erythroid 2-related factor 2 (NRF2), peroxisome proliferator-activated receptor gamma (PPAR-γ), and steroids, have demonstrated neuroprotective effects in in vivo or in vitro PD models. Clinical trials applying objective biomarkers are required to investigate the therapeutic potential of anti-inflammatory medications for PD.

## 1. Introduction

Parkinson’s disease (PD) is a common neurodegenerative disease characterized by the loss of dopaminergic (DAergic) neurons in the substantia nigra pars compacta (SNpc). Its main symptoms include resting tremors, rigidity, shuffling gait, and bradykinesia. The pathogenesis of neurodegeneration in PD is driven by the abnormal accumulation of misfolded α-synuclein in the central nervous system (CNS) [1]. The subsequent neurotoxic cascades involving genetic [2,3], environmental [4], and immunological factors [5] can further enhance the neurotoxicity of misfolded α-synuclein, causing neurodegeneration in the neighboring brain regions. Genome-wide association studies have identified many genetic variants associated with PD. Studies of animal models, neuroimages, and postmortem pathology have also provided substantial insights into the involvement of neuroinflammation in PD pathogenesis [6,7,8], and indicate that cytokine-induced inflammatory responses may play a vital role.

At present, no effective treatment exists to halt PD progression. Sensitive and practical biomarkers of PD are urgently required, and their efficacy for diagnosing PD in early or presymptomatic stages should be validated in clinical trials. Various molecules in the cerebrospinal fluid (CSF), such as α-synuclein, DJ-1, amyloid-β, tau, and lysosomal enzymes, may be biomarkers of PD [9,10]. Positron emission tomography, single-photon emission computed tomography, and magnetic resonance imaging are important imaging tools that reveal DAergic nerve projections in SN. Recently, studies involving neuroimaging, neuropathology, and cell and animal models further indicated an important interaction between neuroinflammation and neurodegeneration of DAergic neurons in PD [6,7,8]. Here, we review findings from key studies on the inflammatory biomarkers of PD and further examine the role of these biomarkers in systemic and brain inflammatory responses in PD pathogenesis.

## 2. Role of Neuroinflammation in PD

In the early 1980s, McGeer observed activated microglial infiltrations in the SN of the postmortem PD brain [6]. Numerous studies have since been conducted on the neuroinflammation associated with PD pathogenesis, such as increased proinflammatory cytokines in the blood [11] or CSF [10,12]. Activated microglia secrete several proinflammatory cytokines, such as tumor necrosis factor-α (TNF-α), interleukin (IL)-1β, and IL-6 [11,13,14,15,16]. They also express major histocompatibility complex (MHC) class II, and are associated with damaged neurons in patients with PD [17]. Furthermore, neuroimaging studies have used radiotracers specific to microglial activation to demonstrate ongoing neuroinflammation in PD [18,19]. A well-known example is [11C](R)PK11195 binding to several brain regions in patients with PD [7].

The aggregation of the abnormal, insoluble form of α-synuclein plays a key role in PD pathogenesis [20]. Misfolded α-synuclein is involved in the pathogen-associated molecular pattern- or damage-associated molecular patterns (DAMP)-mediated dysregulation of microglial toll-like receptor (TLR)2 or TLR4-mediated signaling pathway, which ultimately activates myeloid differentiation primary response 88 (MyD88) and nuclear factor kappa B (NFκB), triggering TNF-α and IL-1β production [21]. The treatment of BV2 mouse microglial cells or primary microglia with aggregated α-synuclein upregulates the production of TNF-α, IL-1β, monocyte chemoattractant protein (MCP)-1, and interferon (IFN)-γ [22,23,24]. Panicker et al. demonstrated that aggregated α-synuclein binds to the microglial surface cell membrane receptors TLR2 and CD36, then recruits Fyn kinase, thereby activating and subsequently phosphorylating protein kinase C-delta (PKCδ); this leads to increased PKCδ-dependent activation of the NFκB pathway, followed by increased IL-1β production [25]. Knockout of *TLR2* reduces the uptake of α-synuclein in mouse microglia [26]. The activation of the TLR-4–NFκB pathway mediates the incorporation of α-synuclein into autophagosomes [27,28]. A functional block of TLR4 in BV2 mouse microglia or *TLR4*-knockout primary mouse microglia inhibits the uptake of α-synuclein and prevents TNF-α and IL-6 production [29]. α-Synuclein also increases the microglial expression of IFN-γ, thereby inducing neuronal MHC-I expression; thus, the neurons can be selectively targeted by CD8+ T cells [30]. α-Synuclein is encoded by *SNCA*. SNCA overexpression in rat SN decreases fiber density in DAergic neurons and increases the number of MHC-II+ microglial cells [31]. In 1-methyl-4-phenyl-1,2,3,6-tetrahydropyridine (MPTP)-treated mice, T cells from mice immunized to nitrated α-synuclein potentiate neurodegeneration in response to MPTP [22]. Both proinflammatory type 1 T helper (Th1) and type 17 T helper (Th17) subtypes can enhance MPTP-induced neurodegeneration, whereas the regulator T cell (Treg) subtype is protective against it [32]. These results support the role of T cell subsets activated by α-synuclein-induced immune responses in the pathogenesis of DAergic neurodegeneration.

Humoral adaptive immunity is also involved in PD pathogenesis. Numerous autoantibodies target CNS-specific proteins, such as tau, S100B, glial fibrillar acidic protein (GFAP) [33,34,35], neurofilament [36,37], GM1 [38], and neuronal calcium channels [39]; moreover, autoantibodies to α-synuclein [40,41] have also been discovered. Blood levels of anti-melanin antibody are elevated in the early stages of PD [42]. Together, these findings indicate that both innate and adaptive immune systems are activated in PD.

In addition to the brain, α-synuclein aggregation has also been discovered in the enteric nervous system (ENS) [43]. The expression of α-synuclein in enteric neurites is positively correlated with the degree of intestinal wall inflammation [44,45]. The expression of TNF-α, IFN-γ, IL-6, and IL-1β is upregulated in colon biopsy samples of patients with PD [46]. IL-1 and IL-8 are also elevated in stool specimens of patients with PD [47]. Altered gut metabolites and microbiomes are also involved in intestinal inflammation in patients with PD [48,49,50]. Notably, specific gut metabolites may increase neurodegeneration in PD. In *SNCA* transgenic mice, short-chain fatty acids produced by the intestinal microbiome lead to a higher degree of α-synuclein aggregation in the basal ganglia and SN, potentiating motor deficits [47]. Fecal microbiota transplantation in 11 PD patients with constipation increased the abundance of *Blautia* and *Prevotella* in feces and improved motor and nonmotor symptoms [51]. Therefore, PD pathogenesis likely involves an interplay among gut microbiota, metabolites, and cytokines.

## 3. Candidate Biomarkers of Inflammation in PDs

The clinical diagnosis of PD is made mostly based on clinical symptoms, which may appear only in advanced disease stages, thus precluding therapeutic intervention in early stages. Biomarkers are important for detecting PD in the early stage as well as for monitoring disease progression and treatment responses. Among molecular biomarkers of PD, α-synuclein, tau, and Aβ42 in the CSF, blood, and other body fluids have attracted considerable research interest [10,52,53,54,55,56,57]. Inflammatory molecules can be used as potential biomarkers to reflect the neuroinflammatory pathogenesis of PD [10,15,58]. Because obtaining live human neurons from patients with PD is challenging, the CSF is an acceptable source and can be used to detect molecular changes underlying the neurodegenerative pathogenesis. The leakage of inflammatory factors from degenerated brain regions can also be detected in the peripheral blood. The alterations of inflammatory biomarkers in the blood of patients with PD also indicate the peripheral involvement of PD pathogenesis, such as the gut–brain axis. Recent studies have described IL-1β, IL-2, IL-6, IL-10, high-sensitivity C-reactive protein (hsCRP), TNF-α/soluble TNF-receptors (sTNFRs), and regulated upon activation, normal T cell expressed and presumably secreted (RANTES), as potential peripheral biomarkers (Table 1).

### 3.1. IL-1β

IL-1β is a proinflammatory cytokine with pleiotropic biological actions in the peripheral blood and brain. Sustained IL-1β expression in the striatum causes DAergic neuronal death and motor disabilities in rats [59]. IL-1β levels are elevated in the striatum of patients with PD [60,61]. IL-1β levels in the CSF are elevated in patients with PD, particularly those with probable REM sleep behavior disorder (PRBD) [62]. Serum IL-1β levels are significantly elevated in patients with PD, and those who also exhibit high titers of antibodies against common pathogens [63,64]. A large multicenter study demonstrated higher serum IL-1β levels in patients with PD compared with control participants [11]. However, other studies did not observe alterations in IL-1β levels in the serum [65] and CSF [66] samples of patients with PD. A 2016 meta-analysis including six studies (623 patients) concluded that blood IL-1β levels are elevated in patients with PD [15].

### 3.2. IL-2

The gut microbiome composition may alter cytokine profiles and affect inflammatory processes in PD [67], whereas IL-2 can suppress chronic inflammation in the gastrointestinal tract [68,69,70]. IL-2 plays a critical role in T cell proliferation, Treg cell expansion, and mediation of inflammation-induced cell death [71]. Decreased blood IL-2 levels reduce the number and function of Treg cells, leading to lymphoproliferation and autoimmunity [71]. IL-2 levels are elevated in the striatum of patients with PD [72]. Patients with PD have higher serum IL-2 levels than control participants [11,73,74]; the higher serum IL-2 levels can be reduced by treatment with antiparkinsonian medications [74]. In addition, high serum levels of soluble IL-2 receptors (sIL-2R) are associated with severe symptoms of anxiety or depression in patients with PD [75]. The meta-analysis in 2016 including three studies (282 patients) revealed the elevation of IL-2 in the blood of patients with PD [15].

### 3.3. IL-6

IL-6 is a multifunctional cytokine mainly secreted by neurons and glial cells, and it plays a vital role in neuronal development and differentiation [76]. It triggers neuronal survival after injury but also causes neuronal death in neurodegenerative diseases [77]. IL-6 levels are elevated in the striatum, CSF, and serum of patients with PD [64,73,75,78,79,80,81,82,83]. Higher serum IL-6 levels are correlated with infection in patients with PD [63]. Serum IL-6 levels are inversely correlated with clinical parameters, including functional mobility, gait speed, and Mini-Mental Status Examination scores, in patients with PD [84,85]. Scalzo et al. reported that serum IL-6 levels cannot reflect PD severity because serum IL-6 levels were not correlated with the scores of Unified Parkinson’s Disease Rating Scale (UPDRS) part III and H&Y stage [84]. However, regarding the nonmotor symptoms of PD evaluated using UPDRS part I, plasma IL-6 levels were correlated with the severity of depression [85]. Another study reported no correlation of serum IL-6 levels with H&Y stages, disease duration, and UPDRS scores [81]. Elevated serum IL-6 levels are also associated with death in patients with PD [86]. The scores of the activity daily living scale in patients with PD are negatively correlated with serum IL-6 levels [13]. However, some studies have not detected an elevation of serum IL-6 levels in patients with PD [11,64,66,74], although a 2016 meta-analysis including 13 studies (898 patients) revealed higher peripheral IL-6 levels in patients with PD [15].

### 3.4. IL-10

IL-10 is an anti-inflammatory cytokine produced by lymphocytes and microglia [87]. It has neuroprotective effects against LPS-induced cell death [88]. Serum IL-10 levels are increased in patients with PD compared with control participants [11,73,89]. However, two studies have not indicated any changes in serum and CSF IL-10 levels in patients with PD [66,90], whereas the meta-analysis in 2016 including five studies (376 patients) demonstrated higher peripheral IL-10 levels in patients with PD [15].

### 3.5. TNF-α/sTNFRs

TNF-α is a proinflammatory cytokine that plays a key role in host defense [91]. TNF-α binds to sTNFR and regulates sTNFR expression; sTNFR expression may be an indicator of TNF-α activity [92]. TNF-α activates microglia to induce the progressive loss of DAergic neurons in the SN [93,94,95]. TNF-α is upregulated in the SN of patients with PD [96]. TNF-α levels in the CSF are elevated in PD patients [94], particularly those with PRBD [62]. Serum TNF-α levels are also elevated in patients with PD [11,66,73,75,82,83] and those with atypical parkinsonism [73]. Elevated plasma sTNFR1 is associated with poor executive function in patients with PD [97]. Plasma TNF-α levels are positively correlated with cognitive impairment, depression, and disability in patients with PD [75,98]. Serum TNF-α levels are not significantly elevated in PD patients with infectious burdens [63]. The meta-analysis in 2016 including nine studies (809 patients) demonstrated higher peripheral TNF-α levels in patients with PD [15].

### 3.6. RANTES

RANTES is a proinflammatory chemokine involved in the regulation of immunoreactions and the recruitment of immune cells such as monocytes, granulocytes, and T cells to sites of inflammation [99]. A study reported that serum RANTES levels in patients with PD were higher than those in control participants [100]. Serum RANTES levels are positively correlated with H&Y stages and disease duration [82,101], but are not associated with UPDRS scores [82]. However, Gangemi et al. noted that serum RANTES levels were comparable in patients with PD and control participants [102], whereas the meta-analysis in 2016 including five studies (171 patients) demonstrated higher blood RANTES levels in patients with PD [15].

### 3.7. High-Sensitivity C-Reactive Protein (hsCRP)

The circulating hsCRP level is a useful marker of ongoing inflammation or tissue damage [103]. hsCRP has potential as a marker of neuroinflammation in PD [104]. Elevated plasma hsCRP levels are present in patients with PD who underwent levodopa treatment [105]. Serum hsCRP levels are also higher in patients with PD than in control participants [106,107]. However, these elevations of hsCRP have not been recapitulated by other studies [11,75,108]. The meta-analysis in 2016 including six studies (696 patients) demonstrated higher blood hsCRP levels in patients with PD [15].

## 4. Genetic Mutations Involved in Neuroinflammation in PD

In addition to *SNCA*, the roles of other PD-causative genes such as *PINK1*, *PRKN*, *DJ-1*, and *LRRK2* [109] have been demonstrated in neuroinflammation.

### 4.1. Leucine-Rich Repeat Kinase 2

Mutations in leucine-rich repeat kinase 2 (LRRK2) are the most common monogenic genetic causes of both familial and sporadic PD [110,111], and they are also present in other inflammatory diseases such as Crohn’s disease and leprosy [112,113]. LRRK2 expression and kinase activity are upregulated in lipopolysaccharide (LPS)-activated rat microglia, whereas the inhibition of LRRK2 kinase reduces the secretion of TNF-α [114]. Increased secretion of IL-1β and IL-6 is also noted in LPS-activated microglia derived from *LRRK* (p.R1441G) transgenic mice [115]. These findings support the role of LRRK2 in neuroinflammation in PD [115]. *LRRK2* (p.G2019S) transgenic rats demonstrate increased microglial activation in the SN and pronounced DAergic neurodegeneration in response to the overexpression of α-synuclein [116]. Neuroinflammation associated with the *LRRK2* (p.G2019S) mutation could be diminished by LRRK2 kinase inhibition [116]. A study showed that microglia from *LRRK2* (p.G2019S) transgenic mice demonstrate increased expression of IL-6 and TNFα, following injection with recombinant α-synuclein fibrils [117]. Chronic dextran sodium sulfate-induced colitis aggravates microglial activation, loss of DAergic neurons, and locomotor deficits in *LRRK2* (p.G2019S) transgenic mice, whereas treatment with anti-TNF-α antibody attenuates neuroinflammation and neurodegeneration [118]. In *LRRK2* (p.G2019S) knockin mice treated with LPS, depletion of microglia by PLX-3397 diminishes weight loss and increases home-cage activity [119], supporting the interaction between neuroinflammation and LRRK2-mediated neurodegeneration.

### 4.2. PTEN-Induced Putative Kinase 1

Mutations in PTEN-induced putative kinase 1 (*PINK1)* are linked to familial PD with autosomal recessive inheritance [120,121,122]. PINK1 senses mitochondrial dysfunction and phosphorylates parkin to degrade damaged mitochondria through mitophagy [123,124]. PINK1 is also involved in the regulation of proinflammatory cytokines. *PINK1* knockout mice demonstrate increased striatal IL-1β levels, IL-12, and IL-10 after treatment with LPS [125]. In the cortical slices of *PINK1* knockout mice, LPS also augments the upregulation of TNF-α, IL-1β, and IL-6 levels [126]. Moreover, mitochondrial stress leads to the release of DAMPs to activate inflammation, whereas mitophagy mitigates inflammation by removing the damaged mitochondria [127,128,129,130,131]. These results support the role of PINK1-mediated and parkin-mediated mitophagy in inhibiting neuroinflammation.

### 4.3. Parkin (PRKN)

Mutations in *PRKN* are commonly seen in patients with autosomal recessive early-onset PD [132,133,134]. *PRKN* encodes an E3 ubiquitin ligase (parkin), which plays a neuroprotective role against α-synuclein toxicity and oxidative stress [135,136]. Together with PINK1, parkin participates in mitophagy to degrade damaged mitochondria. The nigral DAergic neurons in *PRKN* knockout mice are vulnerable to LPS-induced inflammation [137]. LPS and TNF-α also downregulate parkin expression in BV2 mouse microglial cells [138], suggesting that chronic inflammation modulates *PRKN* expression.

### 4.4. DJ-1

Mutations in *DJ-1* are found in the familial recessive form of PD [139]. These mutations disturb the function of the protein in the regulation of membrane receptor tracking and signal transduction [140], TLR3/4 mediated endocytosis [140], and production of IL-6 and IL-1β [140,141]. In BV2 mouse microglial cells, DJ-1 binds to the p65 subunit of NFκB, and DJ-1 knockdown promotes p65 nuclear translocation [142]. *DJ-1* knockout mice exhibit profound microglial activation compared with wild-type littermate controls, especially in response to LPS treatment [142]. *DJ-1* knockdown in N9 mouse microglial cells also reduces the expression of triggering receptors on myeloid cells 2 (TREM2), which is a pivotal regulator of proinflammatory cytokines such as IL-1β and IL-6 [143].

## 5. Radiotracers Targeting Microglial Activation

Microgliosis is the hallmark of neuroinflammation [144,145]. Postmortem studies have indicated that microglia mediate immunity and initiate neuroinflammation in PD [6]. Many researchers have been trying to identify imaging markers specific to activated microglia to detect PD at an early stage. Radiotracers targeting inflammatory cells can help monitor the neuroinflammatory process in patients with PD [19,146]. Translocator protein (TSPO) is a mitochondrial translocator protein that is highly expressed in activated microglia [19]. The binding levels of [^11^C]PK11195, the first TSPO ligand, are positively correlated with the severity of motor dysfunction and inversely correlated with dopamine transporter markers [11C] 2-B-carbomethoxy-3B-(4-fluorophenyl) tropane ([^11^C]CFT) [147]. However, its binding is not specific to the nigrostriatal regions, and such binding can also be found in the pons, basal ganglia, and frontal and temporal cortices [19]. A second-generation TSPO tracer, [18F]-FEPPA, was developed to detect neuroinflammation specific to the striatum [148]. It demonstrated superior specificity to [^11^C]PK11195 in the striatum in 6-hydroxdopamine (6-OHDA)-treated rats [149]. Large-scale human studies are warranted to validate these findings before their clinical application.

## 6. Anti-Inflammation Strategies for PDs

Molecular and neuroimaging studies have indicated the role of neuroinflammation in PD pathogenesis. Therefore, anti-inflammatory therapies may be a strategy against neurodegeneration in PD. Different anti-inflammatory strategies, including nonsteroid anti-inflammatory drugs (NSAIDs), inhibitors of TNF-α and NLR family pyrin domain containing 3 (NLRP3), agonists of nuclear factor erythroid 2-related factor 2 (NRF2), and peroxisome proliferator-activated receptor (PPAR)-γ, have been studied for treating PD (Table 2).

### 6.1. NSAIDs

In addition to inhibiting cyclooxygenase, NSAIDs downregulate the expression of the deactivate nonsteroidal anti-inflammatory drug-activated gene-1 to suppress microglial activation [150]. In MPTP-treated mice, sodium salicylate decreases microglial activity and lymphocyte infiltrations, and reduces the death of DAergic neurons in SN [151,152,153]. Ibuprofen and piroxicam protect DAergic neurons in SN against rotenone-induced toxicity in rats [154]. Aspirin, acetaminophen, and ibuprofen protect DAergic neurons against glutamate-mediated excitotoxicity in a rat embryonic mesencephalon neuronal model [155]. These animal studies have indicated that NSAIDs may preserve neuronal integrity and survival [155]. However, epidemiological studies have shown no association between ibuprofen or acetaminophen and PD [156]. Neither meta-analysis nor observational studies have provided solid evidence that NSAIDs decrease the risk of PD or modify disease progression [157,158]. Further studies are required to verify the protective role of NSAIDs in patients with PD.

### 6.2. TNF-α Inhibitor

MPTP administration upregulates TNF-α expression in mouse striatum preceding the loss of DAergic neurons [151], suggesting the role of TNF-α in preclinical or early-stage PD. MPTP-induced loss of DAergic neurons is abolished in transgenic mice carrying homozygous mutant alleles for *TNFRs* [151]. Thalidomide, an inhibitor of TNF-α synthesis, and *TNF-α* knockout attenuate MPTP-induced neuronal damage in the mouse striatum [159]. A cohort study reported that early exposure to anti-TNF therapy is associated with reduced PD incidence [160]. In this study, patients with inflammatory bowel disease (IBD) were 28% more likely to develop PD than matched individuals without IBD. Patients who are exposed to anti-TNF therapy show a 78% reduction in PD incidence compared with unexposed patients [160]. Although the study has positive results, anti-TNF compounds may have limited CNS effects due to their poor penetration across the blood–brain barrier [161].

### 6.3. NLRP3 Inhibitor

α-Synuclein binds to TLR2 to activate the NLRP3 inflammasome and its downstream IL-1β pathway [162]. A pathological study showed the upregulation of NLRP3 colocalized with microglia in the SN of patients with PD [163]. The small-molecule NLRP3 inhibitor MCC950 decreases inflammasome activation and effectively mitigates motor deficits, nigrostriatal DAergic degeneration, and accumulation of α-synuclein aggregates in 6-hydroxydopa- and α-synuclein fibrils-treated mice [163]. These observations suggest that NLRP3 persistently promotes neuroinflammation, driving progressive DAergic neuropathology, highlighting its potential as a target for PD treatment [163].

### 6.4. NRF2 Enhancer

NRF2 is a transcription factor that regulates endogenous antioxidative and anti-inflammatory pathways [164]. Neuroinflammation is a prominent cause of oxidative stress in PD [165]. Therefore, the reduction of oxidative stress and neuroinflammation by NRF2 enhancers could be a therapeutic strategy for PD. Dimethyl fumarate, a well-known medication in multiple sclerosis, is a potent NRF2 enhancer that reduces the production of reactive oxygen species in the neurons of *SNCA* (p.A53T) transgenic mice [166]. Dimethyl fumarate also prevents nigral DAergic neuron damage and decreases microgliosis in MPTP- and α-synuclein-treated mice [167,168]. These findings suggest that NRF2 is a viable target for therapeutic interventions in PD.

### 6.5. PPAR-γ Agonist

PPAR-γ is a member of the nuclear receptor superfamily that regulates mitochondrial function and modulates lipid and glucose metabolism [169]. PPAR-γ agonists, such as pioglitazone, reduce inflammation by inhibiting the expression of IL-6 and TNF-α [170]. Pioglitazone attenuates inflammatory responses and preserves DAergic nigrostriatal function in the brain of MPTP-treated monkeys [171]. Furthermore, administration of pioglitazone attenuates MPTP-induced glial activation and prevents the loss of dopaminergic neurons in SN of MPTP-treated mice [172,173]. Another PPAR-γ agonist, rosiglitazone, also prevents the loss of DAergic neurons in the SN of MPTP-treated mice [174]. These results support the application of PPAR-γ agonists as putative anti-inflammatory therapies for halting PD progression.

### 6.6. Steroid Drugs

Dexamethasone, a well-known anti-inflammation agent, protects nigral DAergic neurons against LPS-induced toxicity [175]. Steroid precursors such as dehydroepiandrosterone (DHEA) and pregnenolone provide another treatment option for PD [176]. Pregnenolone alleviates synaptic defects and hyperdopaminergic activity in rats [177]. In MPTP-treated monkeys, DHEA improves parkinsonian phenotypes and potentiates the effect of L-dopa [178]. A recent cohort study indicated that dexamethasone was associated with decreased odds of PD, suggesting that corticosteroids are a potential disease-modifying drug in PD [179].

The aforementioned findings indicate the potential of anti-inflammatory therapies for treating PD. These results should be validated by large randomized controlled trials in patients with PD.

## 7. Conclusions

PD pathogenesis is complex and still not fully understood. Neuroinflammation exacerbates DAergic neurodegeneration. This inflammatory cascade involves microglial activation and marked secretion of proinflammatory cytokines. Tracing the alterations of proinflammatory biomarkers, such as IL-1β, IL-6, IL-10, TNF-α, RANTES, and hsCRP, in the CSF or blood can aid in the early diagnosis of PD and monitoring of disease progression. [^11^C]PK11195 and [18F]-FEPPA radiotracers can help detect neuroinflammation in the brain. Together, these findings further our understanding of how neuroinflammation participates in neurodegeneration, suggesting a basis for future drug discoveries. Further studies to validate the potential of proinflammatory biomarker candidates in large and prospective PD cohorts are warranted. Identification of composite biomarkers by machine learning may lead to the development of sensitive panels for the early detection of PD and monitoring disease progression. Randomized controlled trials investigating objective biomarkers should be conducted to determine the therapeutic potential of anti-inflammatory medications for PD.

**Table 1:** Potential biomarkers involved in neuroinflammation in Parkinson’s disease.

Candidate Biomarker	Origin	Change	Correlated Parameters	Reference
IL-1β	Serum	↑PD	UPDRS-III, MMSE	[11]
	Serum	↑PD with IB		[63]
	Serum	↑PD		[64]
	Serum	≈PD		[66]
	CSF	↑PD with PRBD		[62]
	CSF	≈PD		[65]
IL-2	Serum	↑PD	MMSE	[11]
	Serum	↑PD		[73,74]
sIL-2-R	Serum	↑PD		[75]
IL-6	Serum	↑PD	UPDRS-III	[11,78]
	Serum	↑PD		[73,75,79,80,82,83]
	Serum	↑PD with IB		[63]
	Serum	↑PD	CGS, TUG	[84]
	Serum	↑PD with depression		[85]
	Serum	↑PD mortality		[86]
	Serum	↓PD		[64]
	Serum	≈PD		[11,74,76,81]
	CSF	↑PD		[78,80]
IL-10	Serum	↑PD		[11,73,89]
	Serum	≈PD		[66,90]
TNF-α	Serum	↑PD,	UPDRS-III, MMSE	[11]
	Serum	↑PD,		[66,75,82]
	Serum	↑PD		[73]
	Serum	↑PD,	Body sway, Reaction time	[83]
	Serum	≈PD with IB		[63]
	CSF	↑PD,		[94]
	CSF	↑PD with PRBD		[62]
sTNFR1	Serum	↑PD	MMSE, Programming task of FAB	[97]
RANTES	Serum	↑PD		[82,100,101,102]
	Serum	↑PD	H&Y, disease duration	[81]
hsCRP	Serum	↑PD		[106,107]
	Plasma	↑PD		[105]
	Serum	≈PD	UPDRS-III, MMSE	[11]
	Serum	≈PD		[75,108]

↑: upregulation; ≈: no change; ↓: downregulation; CGS: Comfortable Gait Speed; FAB: Frontal Assessment Battery; hsCRP: high-sensitivity C-reactive protein; H&Y: Hohn and Yahr Stage; IB: infectious burden; IL: interleukin; sIL-2R: soluble IL-2 receptor; MMSE: Mini-Mental State Examination; PD: Parkinson disease; PRBD: probable REM sleep behavior disorder; RANTES: Regulated Upon Activation, Normal T Cell Expressed And Presumably Secreted; TNF-α: tumor necrosis factor α; sTNFR: soluble TNF receptor. TUG: timed up and go test; UPDRS-III: Unified Parkinson Disease Rating Scale-Part III.

**Table 2:** Therapeutic target of neuroinflammation in PD.

Target	Medication	Model	Effect	References
COX-inhibitor	Sodium salicylate	MPTP-treated mice	Beneficial	[151,152,153,155]
	Ibuprofen, piroxicam	Rotenone-treated rats	Beneficial	[154]
TNF-α inhibitor	Thalidomide	MPTP-treated mice	Beneficial	[159]
NLRP3 inhibitor	MCC950	6-OHDA-treated mice	Beneficial	[163]
		α-Synuclein fibrils-treated mice	Beneficial	[163]
NRF2 enhancer	Dimethyl fumarate	*SNCA* (p.A53T) transgenic mice	Beneficial	[164]
		MPTP-treated mice	Beneficial	[15]
		Mice expressing α-synuclein in ventral midbrain	Beneficial	[167,168]
PPAR-γ agonist	Pioglitazone	MPTP-treated monkey	Beneficial	[171]
		MPTP-treated mice	Beneficial	[172,173]
	Rosiglitazone	MPTP-treated mice	Beneficial	[174]
Steroidal drugs	Dexamethasone	LPS-treated rat	Beneficial	[175]

COX: cyclooxygenase; LPS: lipopolysaccharide; MPTP: 1-methyl-4-phenyl-1,2,3,6-tetrahydropyridine; NLRP3: NLR family pyrin domain containing 3; NRF2: nuclear factor erythroid 2 related factor 2; 6-OHDA: 6-hydroxydopamine; PPAR-γ: peroxisome proliferator-activated receptor γ; TNF-α: tumor necrosis factor-α.

## Data Availability

No new data were created or analyzed in this study. Data sharing is not applicable to this article.
